# Functionalized Carbon Nanotubes in the Brain: Cellular Internalization and Neuroinflammatory Responses

**DOI:** 10.1371/journal.pone.0080964

**Published:** 2013-11-18

**Authors:** Giuseppe Bardi, Antonio Nunes, Lisa Gherardini, Katie Bates, Khuloud T. Al-Jamal, Claire Gaillard, Maurizio Prato, Alberto Bianco, Tommaso Pizzorusso, Kostas Kostarelos

**Affiliations:** 1 CNR Neuroscience Institute, Pisa Area della recerca, Pisa, Italy and Center for MicroBioRobotics, IIT@SSSA, Pontedera, Pisa, Italy; 2 Nanomedicine Laboratory, UCL School of Pharmacy, University College London, London, United Kingdom; 3 CNRS, Institut de Biologie Moléculaire et Cellulaire, UPR 9021 Immunologie et Chimie Thérapeutiques, Strasbourg, France; 4 Department of Chemical and Pharmaceutical Sciences, University of Trieste, Trieste, Italy; 5 Department of Psychology, University of Florence, Area San Salvi, Florence, Italy; Universidad de Castilla-La Mancha, Spain

## Abstract

The potential use of functionalized carbon nanotubes (*f-*CNTs) for drug and gene delivery to the central nervous system (CNS) and as neural substrates makes the understanding of their *in vivo* interactions with the neural tissue essential. The aim of this study was to investigate the interactions between chemically functionalized multi-walled carbon nanotubes (*f*-MWNTs) and the neural tissue following cortical stereotactic administration. Two different *f*-MWNT constructs were used in these studies: shortened (by oxidation) amino-functionalized MWNT (oxMWNT-NH_3_^+^) and amino-functionalized MWNT (MWNT-NH_3_^+^). Parenchymal distribution of the stereotactically injected *f*-MWNTs was assessed by histological examination. Both *f-*MWNT were uptaken by different types of neural tissue cells (microglia, astrocytes and neurons), however different patterns of cellular internalization were observed between the nanotubes. Furthermore, immunohistochemical staining for specific markers of glial cell activation (GFAP and CD11b) was performed and secretion of inflammatory cytokines was investigated using real-time PCR (qRT-PCR). Injections of both *f*-MWNT constructs led to a local and transient induction of inflammatory cytokines at early time points. Oxidation of nanotubes seemed to induce significant levels of GFAP and CD11b over-expression in areas peripheral to the *f*-MWNT injection site. These results highlight the importance of nanotube functionalization on their interaction with brain tissue that is deemed critical for the development nanotube-based vector systems for CNS applications.

## Introduction

Stereotactic surgery is a clinically used strategy for the delivery of therapeutic agents into the brain. Despite the invasive nature of the methodology, it allows precise and direct access to specific areas within the brain [1], thus offering a realistic intervention for the treatment of neurological diseases, such as Parkinson's disease [2] and Huntington's disease [3], [4]. The unique properties of carbon nanotubes (CNTs) make them promising candidates for the design of tools for interventions of the CNS. During the last decade various studies have been published regarding the utilization of nanotube electrical conductivity, mechanical resilience and morphological similarity to neurites, for neurological applications [5], [6]. Previous studies employing various neuronal cell culture models have explored the potential use of CNTs as substrates for neuronal growth, taking advantage of their capacity to integrate with neurons and enhance neuronal functions, such as increased neurite outgrowth and branching [7]–[10]. *f*-CNTs have also been shown to promote the re-establishment of synaptic connections among neuronal populations. From a different perspective, pre-clinical *in vivo* studies have also demonstrated that *f*-CNTs can contribute to therapeutic strategies against CNS-related disorders with complex pathological aetiologies, such as stroke [11], [12], spinal cord injury [13]and glioblastoma [14].

In view of this potential in neurological applications, it is paramount to understand the interactions between CNT and neural tissue cells *in vivo*. We have previously demonstrated the feasibility of stereotactic administration of MWNTs injected directly into the rodent cortex [15]and have also reported the occurrence of nanotube degradative processes taking place [16]. Others have shown that intratumoral injections of CNTs (pristine MWNTs coated with Pluronic F-108 or SWNTs chemically conjugated with polyethylene glycol) using an intracranial glioblastoma model were well-tolerated, eliciting only transient and self-limiting local inflammatory responses [14]. Nevertheless, knowledge regarding the CNT-neural cell interactions *in vivo* and the CNT fate following cellular internalization within the CNS is limited and requires further investigation.

Previous studies from our laboratories had illustrated that amino-functionalized CNT complexed with siRNA were able to lead to functional rehabilitation in an induced stroke model after stereotactic administration [11]. In this study, we set out to interrogate the interactions between these particular *f-*MWNTs in comparison to their carboxylated iterations (that result in shorter nanotubes) with brain tissue *in vivo*. The present study was particularly designed to elucidate further the possible structure-function relationship between *f-*MWNT surface functionalization strategy and the resultant patterns of cellular internalization and responses. Parenchymal distribution and the intracellular localization of *f*-MWNTs were assessed by histological examination and transmission electron microscopy (TEM) two days and two weeks following stereotactic injection. Furthermore, the inflammatory response of the neighbouring neural tissue to the presence of *f*-MWNTs was examined, in particular regarding cytokine expression and glial cell activation. We report that the type of chemical functionalization used to surface-modify the *f-*MWNTs led to significant differences in nanotube localization and distribution patterns within the brain parenchyma, along with distinct differences in cellular uptake and inflammatory reactivity.

## Methods

### Materials

MWNT were purchased from Nanostructured and Amorphous Materials Inc. (Houston, TX; Lot # 1240XH, 95%). Outer average diameter was 20–30 nm, and length between 0.5–1 µm. MWNT-NH_3_^+^, oxMWNT-NH_3_^+^ and oxMWNT-amide-NH_3_^+^ were prepared and fully characterized as recently described elsewhere [19]. Chemicals and solvents were obtained from Sigma-Aldrich (USA) and were used as received. Vectashield fluorescent mounting media was purchased from Vector Laboratories (USA). 400-mesh copper grids coated with formvar/carbon support film for TEM were from Agar Scientific® (UK). Euthatal® was from Rhone Merieux (UK). Rabbit polyclonal anti-glial fibrillary acidic protein (GFAP) primary antibodies were purchased from Dako (USA) and mouse monoclonal anti-CD11b antibodies were bought from BD (USA). Goat anti-rabbit Alexa 488-conjugated goat anti-mouse-Alexa 568 from Molecular Probes (USA). Real-time PCR primers were obtained from Sigma-Aldrich (USA) and the RNA extraction kit; RNeasy Mini Kit from Qiagen. All RT-PCR reagents; cDNA synthesis kit (Biorad iScript cDNA synthesis kit) and the 1× Fast SYBR Green Master Mix were bought from Biorad.

### TEM micrograph of *f-*MWNT aqueous dispersions

*f-*MWNT powder was hydrated in 5% dextrose solution at 1 mg/ml concentration, by bath-sonicating the dispersions for 15–30 minutes. The sonicated *f*-MWNT dispersions (250 µg/ml) were deposited on 400-mesh copper grids coated with formvar/carbon support film (Agar Scientific®, UK) and allowed to dry at room temperature before imaging under TEM (Philips CM10). The images were captured with high-resolution digital camera coupled to the microscope.

### Ethics statement

All experiments were performed in compliance and with prior approval from the United Kingdom Home Office (1989) and Code of Practice for the housing and care of Animals used in Scientific Procedures under a Home Office project license (PPL 80/2296), evaluated by the UCL School of Pharmacy Ethical Review Committee and in agreement with protocols approved by the Italian Ministry for Scientific Research.

### Animal handling and experimentation

Six to eight-weeks old female C57/Bl6 were caged in groups of 4–7 with free access to food and water. A temperature of 19–22 °C was maintained, with a relative humidity of 45–65%, and a 12 h light/dark cycle.

### Stereotactic administration of *f*-MWNT

C57B1/6 mice were anesthetized with Avertin® (0.5 mL/100 g) and mounted on a stereotactic apparatus. Injections of up to 1 µl volume were made at specific stereotactic locations in the motor cortex (latero-lateral(*x*) +0.5 mm, dorso-ventral(*y*) +1.5 mm, rostro-caudal(*z*) 0.7 mm) by means of a glass pipette (30 μm tip diameter) mounted on a motorized (0.1 μm step) three-axis micromanipulator connected to an injector (Sutter Instruments, Novato, CA, USA). During injections, the animals were oxygenated and heated using a blanket with a thermostat to ensure a 37°C rectal temperature. At the end of all surgical procedures, the scalp incision was closed with Mersilk sutures (Ethicon, UK), and the antibiotic gentamicin was topically administered to prevent infections. In these conditions the whole procedure lasted around 20 min, and recovery from anaesthesia occurred after 60–90 min. After recovery, the animals were returned to their home cages, until they were culled at the desired time-point according to the experimental schedule.

### Tissue (brain) sections and transmission electron microscopy

Mice (n = 2–3) were transcardially perfused under terminal anaesthesia (Euthatal®) with 3% glutaraldehyde in 0.5 M cacodylate buffer (pH 7.4). After perfusion, brains were removed, immersed in the perfusate for 2–4 days at 4°C, allowing adequate penetration of the primary fixative. The brains were then transferred to 1 M cacodylate buffer until ready to be processed (TEM or H&E histology). Specimens of 1 mm were cut using brain matrices (Zivvic Instruments®, USA). Each specimen was washed several times with deionised water, submitted to a second fixation with osmium tetroxide (OsO_4_) for 90 min, rinsed with deionised water, and then dehydrated by a series of ethanol grades: 70%, 90%, and 100%. Specimens were infiltrated with propylene oxide, followed by 1∶1 (v/v) propylene oxide:araldite resin and finally were left in neat resin overnight at room temperature. Specimens were then placed at the desired orientation in the embedding moulds and placed in 60°C oven to allow for resin polymerisation for 2–3 days. Semi-thin sections (approximately 0.71 µm thickness) were obtained using an ultramicrotome and SEMI diamond knife (SEMi, Leica, UK). The stained semi-thin sections were stained with toluene blue, rinsed in distilled water and mounted in a slide to be observed under light microscope. TEM was performed on precisely aligned ultra-thin section (approximately 70 nm thickness) obtained by an ultramicrotome and a diamond knife (Diatome 45°, Leica, UK). The sections were collected onto 400 thin copper 3.05 mm grids (Athene grids, Agar Scientific, UK) and observed unstained under TEM (10–15 grids per animal were observed).

### Hematoxylin and eosin staining of brain tissue

For H&E staining (several slides were imaged per animal), specimens of 1 mm were processed for routine histology by the Laboratory Diagnostic Service of the Royal Veterinary College (London, UK). H&E stained sections of 4 µm thickness were observed and examined under light microscope.

### Immunofluorescence (glia and microglia activation studies)

Injected mice were transcardially perfused with ice-cold 4% paraformaldehyde in 0.1 M TBS, pH 7.4. Brains were quickly removed and cryoprotected in 30% sucrose overnight and then 50 µm coronal sections were cut on cryostat and processed for immunofluorescence. To permeabilize cells and block non-specific antibody binding, free-floating paraformaldehyde fixed 50 µm slices were treated for 90 min at room temperature with 0.2% Triton/phosphate buffered saline (PBS) solution (0.1 M) containing 10% fetal bovine serum (FBS). Rabbit polyclonal anti-Glial Fibrillary Acidic Protein (GFAP) primary antibodies (1∶500 dilution) and mouse monoclonal anti-CD11b (1∶400 dilution) were used overnight at 4°C in the 0.2% Triton/10% FBS/PBS solution. The slices were then incubated with goat anti-rabbit Alexa 488-conjugated (1∶400 dilution) and goat anti-mouse-Alexa 568 (1∶400 dilution) in 10% FBS/PBS solution at room temperature for 2 hours. Slices were rinsed extensively in PBS and mounted on glass slides with Vectashield, preparations were cover slipped, sealed with nail polish, and scanned with a Leica TCS-NT confocal microscope (Leica Microsystems) equipped with an argon–krypton laser, at resolutions of 512×512 pixels at 10× magnification. A certain degree of activated glia markers was present in the cells adherent to the injection, for all three types of *f*-MWNTs and control. To quantify the fluorescence we excluded the cells adherent to the injection site as a minimal level of activation is always present by glial cells in contact with a non-self recognized material, such as *f*-MWNTs. Image files were analyzed off-line with MetaMorph (version 5.0r1 MetaMorph; Molecular Devices) to measure fluorescence intensity in an area of 1×0.5 µm surrounding the injection sites. To evaluate the statistical significance of the results, the average ± SEM of 6 slices/mice was calculated and at least 3 mice per treatment were sacrificed in three independent experiments. One-way statistical analysis of variance followed by analysis with the Student-Newman-Keuls method was performed.

In order to further quantify the fluorescence image files were re-analyzed in an area of 1×0.5 μm surrounding the injection sites with ImageJ® software. Histograms of green (GFAP) or red (CD11b) positive pixel were analyzed. We used the tool ImageJ/Analyze/Histogram/List, followed by the sum of the counted pixels. The positive pixels at lower intensity (5% of the total pixels) were excluded in order to avoid background noise. To evaluate the statistical significance of the results, the average ± SEM of 6 slices/mice was calculated and at least 3 mice per treatment were sacrificed in three independent experiments. One-way statistical analysis of variance followed by analysis with the Bonferroni method was performed.

### Quantitative RT-PCR

After the stereotactic injection with 5% dextrose saline solution, LPS positive control, MWNT-NH_3_^+^ or oxMWNT-NH_3_^+^ B57BL/6 mice were sacrificed at the desired time-point (3, 16, 24 hours, 7 and 14 days) and the brains were quickly removed and anatomically dissected. The right hemisphere of the cortex (injection site) was cryopreserved ready for RNA extraction. Brain tissue was homogenised using liquid nitrogen and total RNA was extracted with an RNeasy Mini Kit according to the manufacturer's instructions (Qiagen). The concentration of total RNA was determined by measuring the optical density 260 nm/280 nm ratio. RNA purity was confirmed between the expected values of 1.8–2.0. First strand cDNA was prepared from 1 μg RNA in a total volume of 20 μL using the Biorad iScript cDNA Synthesis Kit. Real-time-PCR was performed using the CFX96™ RealTime-PCR Detection System (BioRad). The reactions contained 1× Fast SYBR® Green Master Mix (Biorad), each primer at 200 nM (Please see **Table S1** for further details) and 1 μL of cDNA from reverse transcription PCR in a 25 μL reaction. After an initial denaturation step at 95 °C for 10 min, amplification was carried out with 40 cycles of denaturation at 95°C for 10 sec, annealing at 60 °C for 30 sec. Amplification was followed by a melting curve analysis to confirm PCR product specificity. No signals were detected in no-template controls. All samples were run in triplicates and the mean value of each triplicate was used for further calculations. Relative expression was calculated using the 2-ΔΔCT method. The quantity of β-actin (housekeeping) transcript in each sample was used to normalize the amount of each primer transcript. Then the normalized values for each primer transcript were compared to the normalized expression in the 5% dextrose control groups to calculate a fold change value.

To evaluate the statistical significance of the results, the average ± standard deviation (SD) of 4 mice per treatment group and per time-point was calculated. One-way statistical analysis of variance (One-way ANOVA) followed by post-hoc analysis with the Bonferroni method was performed.

## Results

### Chemical functionalization and characterization of *f*-MWNTs

**Figure 1:**
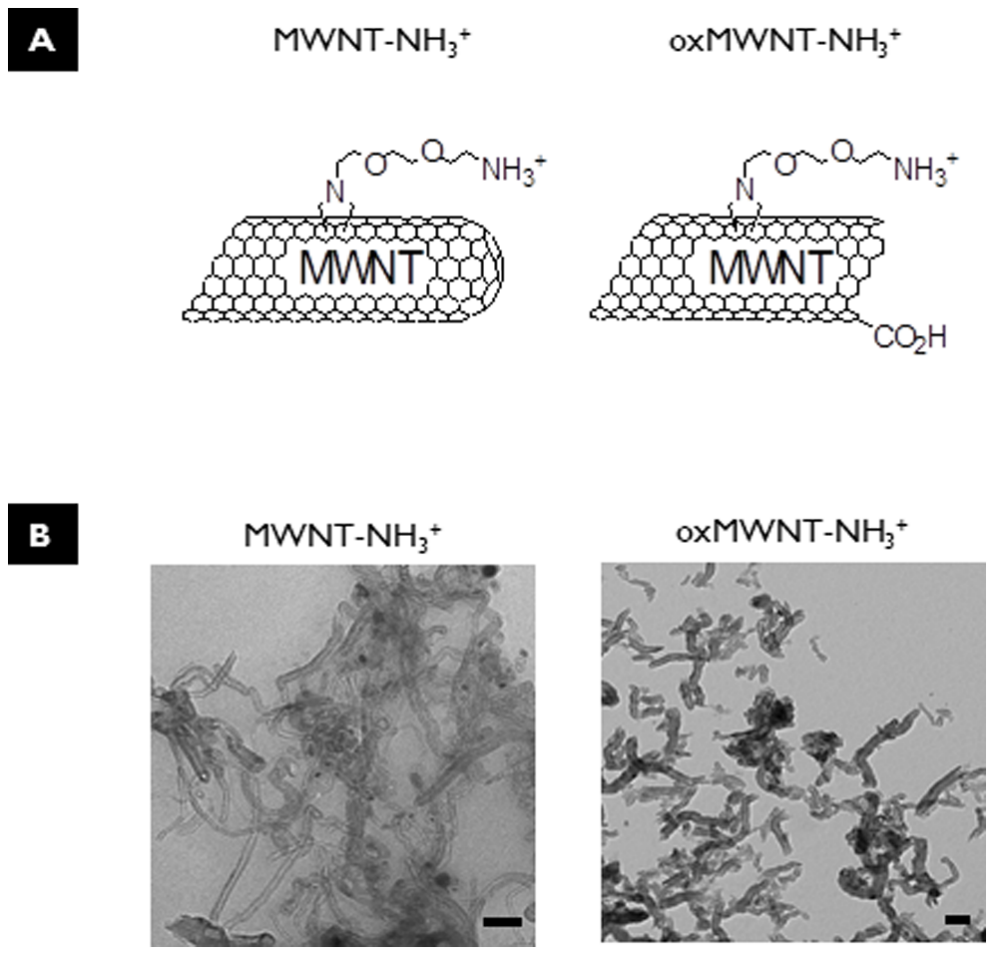
Structural features of *f*-MWNTs. **A**) Chemical structures of *f*-MWNTs. **B**) TEM images of *f*-MWNTs dispersed in 5% dextrose at 250 µg/ml final concentration (scale bar 100 nm).

In order to investigate the effect of chemically functionalized MWNTs on the interactions with cortical brain tissue *in vivo*, two different chemical strategies were used to functionalize the surface of MWNTs: shortened (by oxidization) and amino-functionalized (oxMWNT-NH_3_^+^) and only amino-functionalized (MWNT-NH_3_^+^). MWNT-NH_3_^+^ were functionalized by 1,3-dipolar cycloaddition of azomethine ylides, which we previously reported in Georgakilas et al. [17] and had a length between 0.5–1µm. oxMWNT-NH_3_^+^ were synthesized following a 2-step protocol consisting of: *a*) oxidation of pristine MWNTs, and *b*) introduction of amine groups via 1,3-dipolar cycloaddition reaction, as already reported in the literature [18]. Oxidation of the pristine MWNTs in step (*a*) led to the introduction of carboxyl groups and the shortening of the nanotubes, with lengths between 200–300 nm. The molecular structures and TEM images of both *f*-MWNTs are shown in **Figure 1** **.** TEM analysis indicated that both *f-*MWNTs were individualized, with good aqueous dispersibility, and free from any major impurities. The degree of amino group loading on the *f*-MWNT surface was determined by the Kaiser test and was comparable between both *f*-MWNT (147µmol/g for MWNT-NH_3_^+^ and 170 µmol/g for oxMWNT-NH_3_^+^), while more complete characterization data of the same material has been recently published elsewhere [19].

### *In vivo* brain parenchyma distribution after stereotactic administration of f-MWNTs

**Figure 2:**
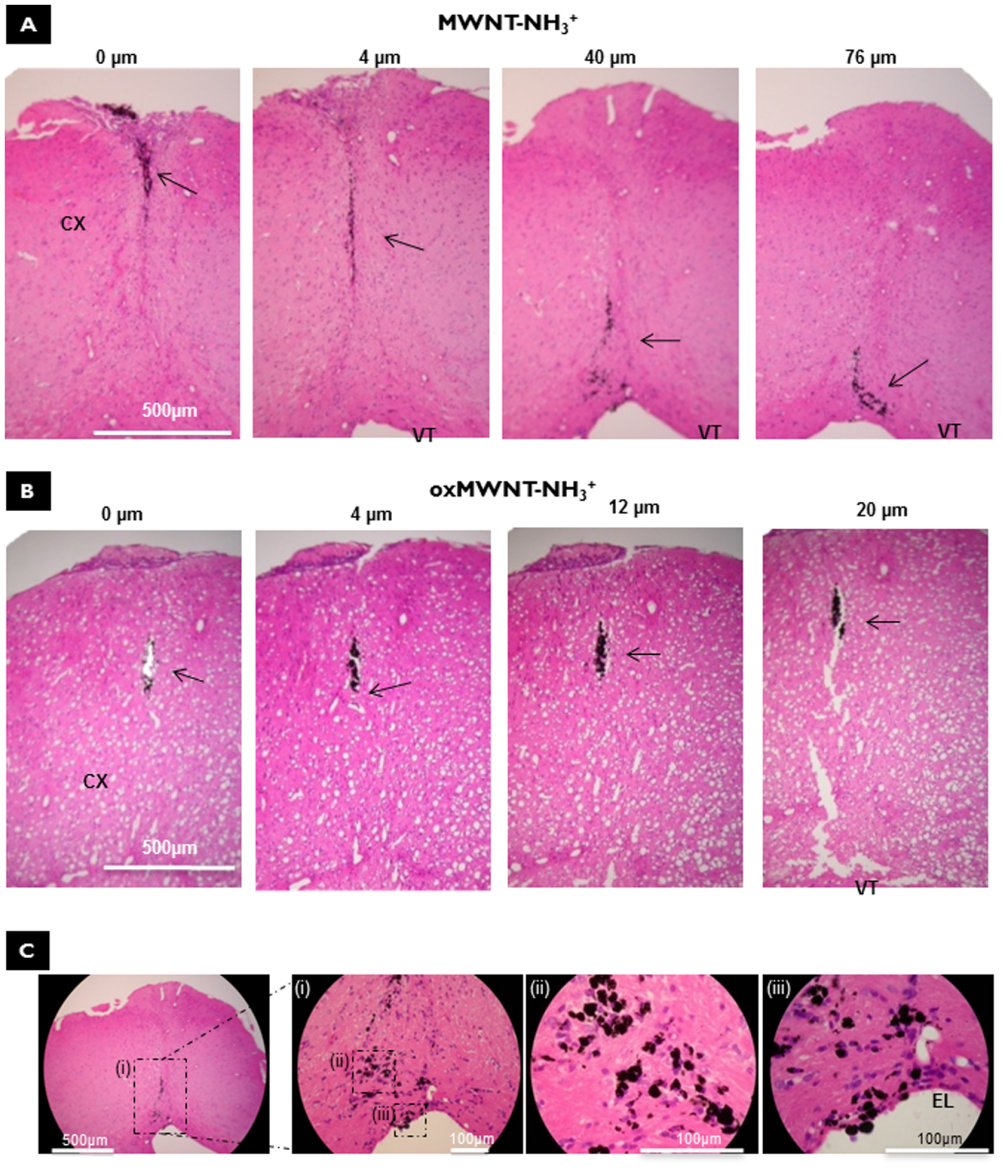
Parenchymal distribution of MWNT-NH_3_^+^and oxMWNT-NH_3_^+^ after stereotactic administration in the motor cortex of C57BL/6 mice by light microscopy of H&E stained coronal sections, two weeks after injection with nanotubes. A) MWNT-NH_3_^+^; B) oxMWNT-NH_3_^+^; C) High magnification images of the MWNT-NH_3_^+^ injected tissue. Cortex (CX), ependymal layer (EL), and ventricles (VT) are noted, respectively. The distance from the Bregma line for each section is indicated above each image. Black arrows indicate the presence of *f*-MWNTs in the sections.

f-MWNTs were stereotactically injected into the motor cortex (latero-lateral(x) +0.5 mm, dorso-ventral(y) +1.5 mm, rostro-caudal(z) 0.7 mm) as a single dose of 500 ng/mouse. Two days and two weeks post-injection, brain tissue was extracted and the parenchymal distribution of MWNT-NH_3_^+^ and oxMWNT-NH_3_^+^ were assessed by performing haematoxylin and eosin (H&E) and by TEM. Histological examination of sequential coronal brain sections at two days post-injection, indicated that both f-MWNT constructs showed no major differences regarding their spatial distribution in the brain parenchyma. Taking into account the number of sequential sections and the thickness of each section, we could observe that oxMWNT-NH_3_^+^ was detected within 310–330 µm in comparison to MWNT-NH_3_^+^ that was detected within 260–270 µm thickness (data not shown). However, at two weeks post-injection, the examination of sequential coronal sections (H&E staining) showed different distribution patterns between MWNT-NH_3_^+^ and oxMWNT-NH_3_^+^ in relation to the injection site (**Figure 2**).

MWNT-NH_3_^+^ were detected more abundantly distributed throughout the injection site and within the brain parenchyma in comparison to oxMWNT-NH_3_^+^. MWNT-NH_3_^+^ were identified in several sequential coronal sections (120–140µm thick) (**Figure 2A**, arrows), whereas oxMWNT-NH_3_^+^ were only observed in a much narrower area, a few sequential sections between 30–50 µm thick (**Figure 2B**, arrows & **Figure S1**). These spatial measurements took into account the dorso-ventral(y) distance between the first coronal section that nanotubes were visualized until the last coronal section where nanotubes could be observed. Furthermore, MWNT-NH_3_^+^ were not only observed local to the injection site, but also seen in different regions of the cortex, in particular close to ependymal cells (that form the interface between the brain parenchyma and ventricles) and also within the brain ventricles (**Figure 2C** & **Figure S2**).

### Internalization of f-MWNT within neural tissue cells following intracortical administration

**Figure 3:**
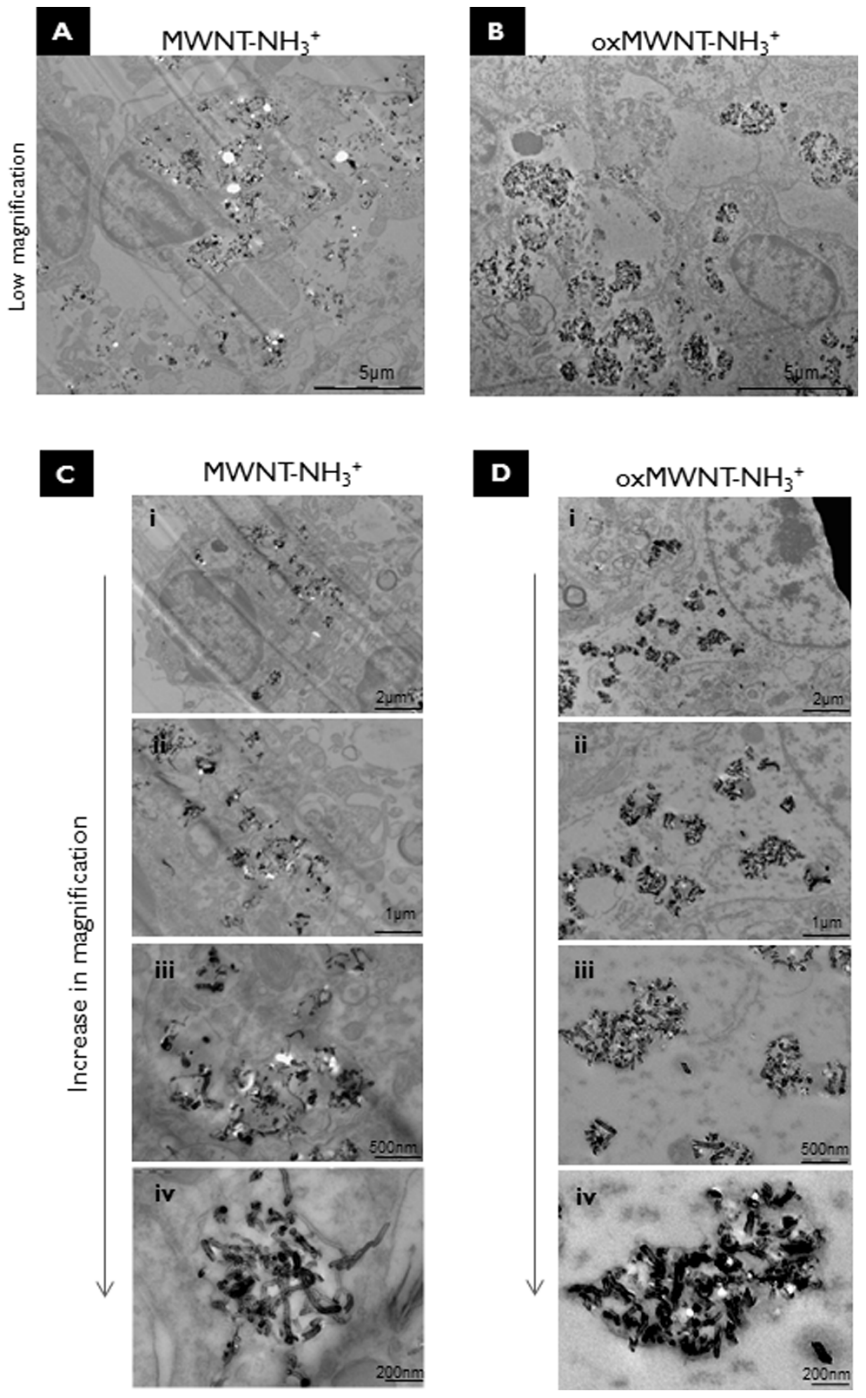
Uptake of *f*-MWNTs by neural tissue cells *in vivo*. Transmission electron micrographs (TEM) of brain parenchyma close to the injection site: **A**) low magnification TEM following injection with MWNT-NH_3_^+^. It was also possible to observe individualized MWNT-NH_3_^+^ at the extracellular spaces. **B**) low magnification TEM following injection with oxMWNT-NH_3_^+^ mainly localized intracellularly in clusters and within vesicles. **C**) and **D**) show TEM images of increasing magnification of a neural tissue cells containing MWNT-NH_3_^+^ and oxMWNT-NH_3_^+^ intracellularly. **i (C & D)** low magnification TEM of a neural cell containing intracellular CNT. **ii and iii (C&D)** shows higher magnification of the cytoplasm of the cell imaged on pannel i (C&D). **iv** shows higher magnification of cytoplasm of cell imaged on panel i, being able to identify the presence of more individualized CNT (**iv C**) or clusters and within vesicle CNT (**iv D**).

To better understand the cellular internalization and intracellular fate of f-MWNTs brain sections were processed and imaged by TEM two days post-injection. Electron micrographs of brain tissue confirmed that both types of f-MWNTs were internalized within neural tissue cells in vivo (**Figure 3**). Interestingly, the two f-MWNT types showed different patterns of intracellular distribution by TEM. Amino-functionalized alone MWNT-NH_3_^+^ were more evenly dispersed throughout the brain parenchyma and within cells (**Figure 3A**). It was possible to observe repeatedly MWNT-NH_3_^+^ being internalized into cells and single, individualized nanotubes piercing cellular membranes (**Figure S3**). Once within the cell, MWNT-NH_3_^+^ appeared either as small aggregates enclosed within membranous intracellular vesicles or as individualized nanotubes residing in the cytoplasm (**Figure 3C**). oxMWNT-NH_3_^+^ on the other hand, despite being shorter than MWNT-NH_3_^+^ were always observed in clusters (**Figure 3B**), predominantly uptaken by neuronal cells as clusters wrapped within intracellular vesicles, presumably microglial phagosomes, very rarely seen individualized in the cytoplasm (**Figure 3D**).

### Cytokine-associated inflammatory response following intracortical administration of f-MWNTs

**Figure 4:**
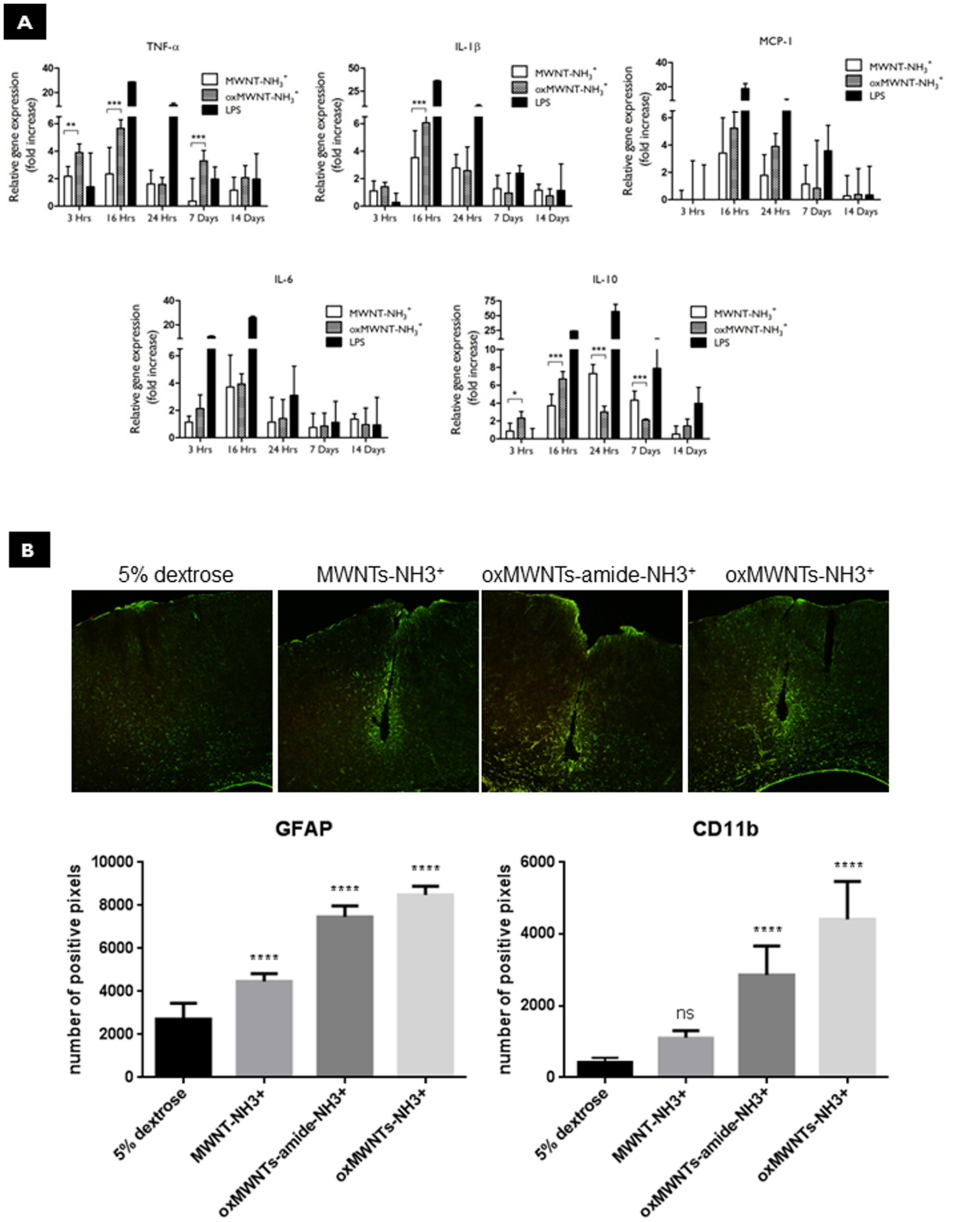
Neuroinflammatory response following intra-cortical injection of *f*-MWNTs. **A**) Panel of cytokine expression profiles in C57BL/6 mouse cortex following stereotactic injection of 5% dextrose solution, LPS, MWNT-NH_3_^+^ and oxMWNT-NH_3_^+^ (*n* = 4 for each group). mRNA expression levels were assessed using quantitative rtPCR. Relative gene expression was calculated using the 2-ΔΔCT method. Relative gene expression levels (plotted as fold increases) were calculated by first normalizing the values for each primer transcript against the house-keeping gene β-actin, and then further normalized against the 5% dextrose control group, giving relative gene expression values (fold-change (mean ± SD)). Both *f*-MWNTs caused a transient increase in the pro-inflammatory cytokine expression levels, peaking at 16 hr. oxMWNT-NH_3_^+^ induced significantly higher expression levels of TNF-α and IL-1β at 16 hr compared to MWNT-NH_3_^+^ (*p*<0.001). **B**) Glia activation studies 30 days after stereotactic injection with 5% dextrose saline solution, MWNT-NH_3_^+^, oxMWNT-amide-NH_3_^+^ and oxMWNT-NH_3_^+^ (*n* = 3 for each group). Histograms of green (GFAP) or red (CD11b) positive pixel were analysed. The positive pixels at lower intensity (5% of the total pixels) were excluded in order to avoid background noise.The intensity was measured and quantified in an area 1×0.5 mm surrounding the injection site.

The induction of inflammatory cytokines (see **Table S1** for further details of primers) in the cortex was then investigated following stereotactic injection of 5% dextrose solution, lipopolysaccharide (LPS) used as positive control, MWNT-NH_3_^+^ and oxMWNT-NH_3_^+^ (**Figure 4A**). All values are expressed as relative levels of mRNA expression at the cortex (including the site of injection) in relation to those following injection with dextrose. LPS induced significant increases in the expression levels of all pro-inflammatory cytokines, greatly exceeding the levels induced by both f-MWNTs, peaking at 16 hr and subsiding by 24 hr. The only anti-inflammatory cytokine that displayed a different profile of expression was IL-10, showing elevated levels up to 7 days and returning to baseline after 14 days. Overall, oxMWNT-NH_3_^+^ consistently induced a higher expression of pro-inflammatory cytokines in comparison to MWNT-NH_3_^+^. At 16 hr post-injection, statistical significance was observed between the two f-MWNTs for the pro-inflammatory cytokines TNF-α and IL-1β. To confirm that the increased expression of cytokines from the f-MWNT injections was transient, we measured cytokine levels at 7 and 14 days. Notably, the increase seen in cortical cytokine expression levels at earlier time points (maximum at 16 hr) decreased to background levels for both f-MWNTs at the later time-points (7 and 14 day). It is important to note that the transient increase in pro-inflammatory cytokine expression levels for both f-MWNTs were only 4-8 times higher than the dextrose injected baseline levels, and significantly lower compared to LPS (20–30 times higher than dextrose saline-injected brain).

### Astrocyte and microglia cell activation following intracortical administration of f-MWNTs

To further explore the potential cellular reactivity by the presence of f-MWNTs in the cortical parenchyma, we investigated the in vivo activation of astrocytes and microglia by immunofluorescence. Coronal brain tissue sections (50µm thickness) were stained with the antibodies GFAP and CD11b, 3 and 30 days post-stereotactic injection. Staining tissue sections for glial fibrillary acidic protein (GFAP) aimed at the identification of activated astrocytes, whereas the microglia/macrophage marker CD11b overexpression was interpreted as a sign of active inflammation [20]. Three days after injection, GFAP and CD11b staining of the control group (5% dextrose saline) was similar to that observed for both f-MWNTs injected groups (**Figure S4** & **Figure S5**). Interestingly, 30 days after stereotactic injection, immunohistochemical analysis revealed that both astrocytes and microglia activation was significantly different between the two types of f-MWNTs (**Figure S4** & **Figure S5**). Semi-quantitative analysis of the histograms of green (GFAP) or red (CD11b) positive pixel in the area around the injection site (1×0.5µm) indicated that the oxMWNT-NH_3_^+^ induced higher expression of GFAP and CD11b (**Figure 4B**). In contrast, injection of MWNT-NH_3_^+^ did not lead to overexpression of astrocyte or microglia activation markers after 30 days post-administration. In order to investigate this phenomena further, we used a third type of chemically functionalized MWNTs that were initially oxidized and then further modified by performing amidation of the carboxylic groups at the surface of those nantoubes using triethylene diamine (oxMWNT-amide-NH_3_^+^) (**Figure S6A**). Similar to oxMWNT-NH_3_^+^, stereotactic injection of the aminated nanotubes oxMWNT-amide-NH_3_^+^ also led to upregulation of GFAP and CD11b positive cells suggesting activation taking place (**Figure 4B** & **Figure S6&C**). It is known and expected that not all carboxylic functions at the nanotube surface can be transformed into ammonium groups. The remaining unreacted –COOH groups at the surface of the oxMWNT-amide-NH_3_^+^ may indeed be provoking the observed inflammatory response. However, further experiments should be undertaken to elucidate such mechanisms in more detail.

Lastly, an observation that was consistent for all brain tissues treated with both types of f-MWNTs that were initially oxidized (oxMWNT-NH_3_^+^ and oxMWNT-amide-NH_3_^+^), but not with the amino-functionalized nanotubes (MWNT-NH_3_^+^), was the presence of mast cells in certain regions of the brain close to perivascular space (**Figure S7**). Mast cells can be identified by metachromatic stain in the presence of blue basic dyes (such as toluidine blue staining) under light microscopy (**Figure S7B**). The ultra-structure of mast cells was also confirmed by TEM (**Figure S7C**). No such evidence of increased numbers of mast cells within brain parenchyma was noted in groups treated with 5% dextrose or MWNT-NH_3_^+^. Mast cells are an important member of both the innate and acquired immune system, and their function as effector cells in inflammatory responses underlie immediate hypersensitivity reactions [21]. Under physiological conditions mast cells are located in restricted areas of the brain (mainly the thalamus and hypothalamus). However, recently it has been shown in an *in vitro* model that mast cells can respond to microglia activation due to cytokine and chemo-attractant release [22].

## Discussion

Even though conflicting data has been reported concerning the safety of CNTs that can lead to confusion, it is important to highlight that chemically functionalized MWNTs have consistently provided a significantly improved safety profile compared to their non-functionalized precursors [23], [24]. Regarding the interactions between CNTs and neural tissue cells *in vivo*, and their impact on potential short- and long-term neurotoxicity, studies remain scarce.

In this work, we attempted to vary the structure and surface characteristics of the carbon nanotubes in order to comparative investigate the relationship between carbon nanotube surface functionalization and cellular reactivity, paying particular attention to the internalization of f-MWNTs, the intracellular fate and the neuroinflammatory response in vivo after exposure to different chemically functionalized nanotubes. One of the nanotubes studied was prepared by the 1,3-dipolar cycloaddition reaction on pristine and purified CNTs that yielded ammonium functionalized MWNTs (MWNT-NH_3_^+^) with a length of between 0.5–1 µm. This type of amino-functionalized nanotubes has been shown to be effective in the delivery of siRNA sequences into solid tumors [25]. More recently, we have reported their capability to deliver siRNA in the rodent cortex and offer therapeutic activity in an endothelin-induced stroke model leading to functional recovery of the behaviour of the animals [11]. The second type of chemically functionalised carbon nanotubes (oxMWNT-NH_3_^+^) investigated in this work also underwent amino-functionalization using the 1,3-dipolar cycloaddition reaction, however the reaction was performed on oxidized MWNTs. This oxidation step both shortened these nanotubes and introduced carboxylic groups on their surface and tips. Previous studies had demonstrated that such type of carbon nanotube is highly biocompatible with neuronal cells *in vitro*, without altering cell viability, neuronal morphology or normal functions in primary neurons [18]. TEM confirmed that both MWNT-NH_3_^+^ and oxMWNT-NH_3_^+^ exhibited adequate aqueous dispersibility and individualization of the tubes that allowed further biological investigations.

Histological examination of sequential coronal sections two weeks after stereotactic injection of nanotubes in the murine cortex revealed that MWNT-NH_3_^+^ were more widely dispersed within brain parenchyma in comparison to oxMWNT-NH_3_^+^. The internalization and intracellular fate of CNTs along with possible neuroinflammatory responses are all of major importance, especially in view of their potential use for the treatment of CNS disorders. The ability of CNTs to internalize within neural cells *in vivo* constitutes an advantageous property as their therapeutic cargo may be directly delivered to the cytoplasm of cells and therefore reach specific intracellular therapeutic targets. Internalization of carbon nanotubes into microglia and neurons *in vivo* has been suggested previously in literature [14], [26]. In order to better visualize the localization and intracellular fate of *f-*MWNTs, TEM examination was performed in unstained sections two days after stereotactic injection into the motor cortex. Results indicated that both carbon nanotubes were taken up by a variety of neural tissue cells (microglia and neurons), with evidence suggesting that microglia were predominantly responsible for the observed cellular uptake. This observation was in agreement with previous studies reporting intratumoral injection of nanotubes in an intracranial glioblastoma model [14]. However, it is important to highlight that in these models the brain tissue is already conditioned by the implantation of the glioblastoma tumor cells, which *per se* affect tissue architecture and the levels of microglia activation.

Once internalized, MWNT-NH_3_^+^ were visualized either within vesicles or as free individualized nanotubes in the cytoplasm. The different intracellular localization sites seem to be related to the prevailing mechanisms of uptake [27]–[28] suggesting a combination of endocytosis or phagocytosis of *f-*MWNT and direct translocation of individualized nanotubes through the cellular membrane. The simultaneous occurrence of internalization pathways has been described in the literature previously using other types of cells [29]–[31]. In agreement with such interpretations, we have recently reported data from two different studies that used non-phagocytic A549 cells or primary human monocyte-derived macrophages (HMMs), a proficient phagocytic cell type [32], [33]. Flow cytometry, confocal microscopy and TEM confirmed that cellular internalization of *f-*MWNT (MWNT-NH_3_^+^ and oxMWNT-NH_3_^+^) can occur simultaneously by multiple internalization pathways [33], the predominant mechanism being dependent on the cell type used (phagocytic or non-phagocytic), as well as on the CNT surface functionalization and physicochemical characteristics of the CNT dispersions. The results in the present study suggest that direct membrane translocation of *f-*MWNT can take place *in vivo* both by piercing the plasma membrane upon nanotube entrance into cells or after vesicular internalisation by piercing the vesicular membrane to escape into the cytoplasm. In both cases, individualization of nanotubes is necessary to facilitate this process. This has been illustrated *in vivo* in this study by comparison of the two nanotube types. TEM showed that MWNT-NH_3_^+^ were mainly individualized and present in both intracellular and extracellular domains, while oxMWNT-NH_3_^+^ were mainly detected as small clusters and within intracellular vesicles, with a very limited amount visualized individual (cluster-free) in the extravesicular cytoplasmic or brain parenchymal areas. Moreover, two weeks after injection oxMWNT-NH_3_^+^ were hardly detectable, suggesting a clearance mechanism in operation that may well involve microglia activation. However, more work is needed on this front to identify such mechanisms.

Any application that involves implantation or injection of material directly into the brain needs to address the question of inflammatory responses triggered following interaction with CNS tissue. In this study, we observed that both *f-*MWNTs induce a transient increase in almost all pro-inflammatory cytokines, yet at significantly lower levels compared to the positive control (LPS). Following injury, reactive gliosis occurs as a response by endogenous glial cells, such reaction is sustained by the activation of both astrocytes and microglia cells, which are key elements in the process of brain inflammation [34], [35]. Reactive gliosis has been suggested as an early marker of damage within the brain, and may be detected morphologically and quantified by measurements of GFAP increase. Microglial cells are the resident immune effectors of the CNS, with the ability to respond to the most subtle neuronal injury [36], [37]. Under certain conditions, microglia become activated, changing to the characteristic macrophage morphology. Following activation, microglia express different proteins and surface markers, such as CD11b, which upon activation is significantly up-regulated [36]. We found that the injection of *f-*MWNT mainly elicited an expression of GFAP and CD11b at the injection site, suggesting a self-limiting local inflammatory response caused by injection of the material. Furthermore, up to 30 days post-injection, only oxidized MWNTs (oxMWNT-NH_3_^+^ or oxMWNT-amide-NH_3_^+^) led to sustained levels of microglia and astrocyte activation at the area around the injection site, implicating the role of carboxyl groups at the nanotube surface. This was not observed in the brains injected with 5% dextrose or MWNT-NH_3_^+^, indicating an improved profile for the latter.

In conclusion, oxMWNT-NH_3_^+^ throughout this study led to cytokine and glial cell activation, suggesting that oxidation of the nanotube surface can contribute to a sustained inflammatory reaction in healthy brain. On the other hand, MWNT-NH_3_^+^ was better tolerated, eliciting only a local and transience inflammatory response, without major activation of glial cells and inflammatory cytokines. The significance of these observations lies on the previously unreported effort to identify the importance of nanotube chemical functionalization on the neurotoxicity of CNT after their interaction with neural cells *in vivo*. Overall, this is deemed extremely important, in particular for the development of both CNT-based delivery systems and implantable devices for CNS applications.

## Supporting Information

Figure S1 Brain distribution of oxMWNT−NH3+ after stereotactic administration in the motor cortex of C57BL/6 mice by light microscopy of H&E stained coronal sections, two weeks after injection with nanotubes. Whole coronal section of the brain (top) shows oxMWNT−NH3+ in the brain parenchyma. Middle and bottom panels show high magnification images of the oxMWNT−NH3+ detected in brain parenchyma. Cortex (CX), and ventricles (VT) are noted, respectively. Black arrows indicate the presence of f-MWNTs in the sections.
(TIF)

Figure S2 Brain distribution of MWNT−NH3+ after stereotactic administration in the motor cortex of C57BL/6 mice by light microscopy of H&E stained coronal sections, two weeks after injection with nanotubes. Parenchymal (left), Ependymal Layer (middle) and Lateral Ventricle (right) regions are shown, depicting the presence of MWNT−NH3+ throughout these regions. Bottom panels show high magnification images of the MWNT−NH3+ injected tissue. Cortex (CX), ependymal layer (EL), and ventricles (VT) are noted, respectively. Black arrows indicate the presence of f-MWNTs in the sections.
(TIF)

Figure S3 Brain cell uptake of MWNT−NH3+ observed by TEM 2 days post cortical administration. The extracellular presence of MWNT−NH3+ in different brain areas (A and B) is shown. It is possible to observe the engulfment process of the CNT in bundles (arrows) or the direct translocation of individualized CNT through the cellular membrane (arrow heads).
(TIF)

Figure S4 Glia activation studies after stereotactic injection. A) & B) Show immunohistochemical staining of brain sections after injection with 5% dextrose solution, MWNT−NH3+ and oxMWNT−NH3+, 3 and 30 days post-injection respectively. Green channel represents glial fibrillary acidic protein (GFAP) positive cells (astrocyte marker) and the red channel represents CD11b positive cells (microglia marker).
(TIF)

Figure S5 Glia activation studies after stereotactic injection with 5% dextrose saline solution, MWNT−NH3+ and oxMWNT−NH3+ (n = 3 for each group). Average fluorescence intensity corresponding to total glia (GFAP) and microglia (CD11b) activation after 3 and 30 days. The intensity was measured and quantified in an area 1×0.5 mm surrounding the injection site.
(TIF)

Figure S6 Effects from cortical injection with oxMWNT−amide−NH3+. A) Chemical structure and TEM images of oxMWNT−amide−NH3+ dispersed in 5% dextrose at 250µg/ml final concentration (scale bar 100 nm). C) & D) Glia activation studies after stereotactic injection with oxMWNT−amid−NH3+. C) Immunohistochemistry of brain sections at 3 and 30 days post-administration. Green channel represents GFAP-positive cells (total glia), red channel represents CD11b-positive cells (microglia). D) Average fluorescence intensity induced by astrocyte activation (GFAP) and by microglia activation (CD11b) after 3 and 30 days, respectively. Intensity was measured and quantified in an area of 1×0.5 mm surrounding the injection sites.
(TIF)

Figure S7 H&E stained light microscopy images of brain sections 14 days post-injections with: A) MWNT−NH3+ and B) oxMWNT−NH3+. H&E staining shows no evidence of the presence of mast cells after stereotactic injection of MWNT−NH3+. Mast cells (arrows) could be visualized after injection of oxMWNT−NH3+ (B and C); C) shows semi-thin section of brain parenchyma 14 days post –injection with oxMWNT−NH3+ stained with toluene blue. Ci) & Cii) represent TEM of mast cells with their typical granules (G) present in the cytoplasm.
(TIF)

Table S1 Sequences of forward and reverse primers used in qRT-PCR.
(TIF)
