# Supplementary figures and images for: Functionalized Carbon Nanotubes in the Brain: Cellular Internalization and Neuroinflammatory Responses
Source: PLoS One. 2013 Nov 18;8(11):e80964. doi: 10.1371/journal.pone.0080964 (PMC3832421; doi:10.1371/journal.pone.0080964)

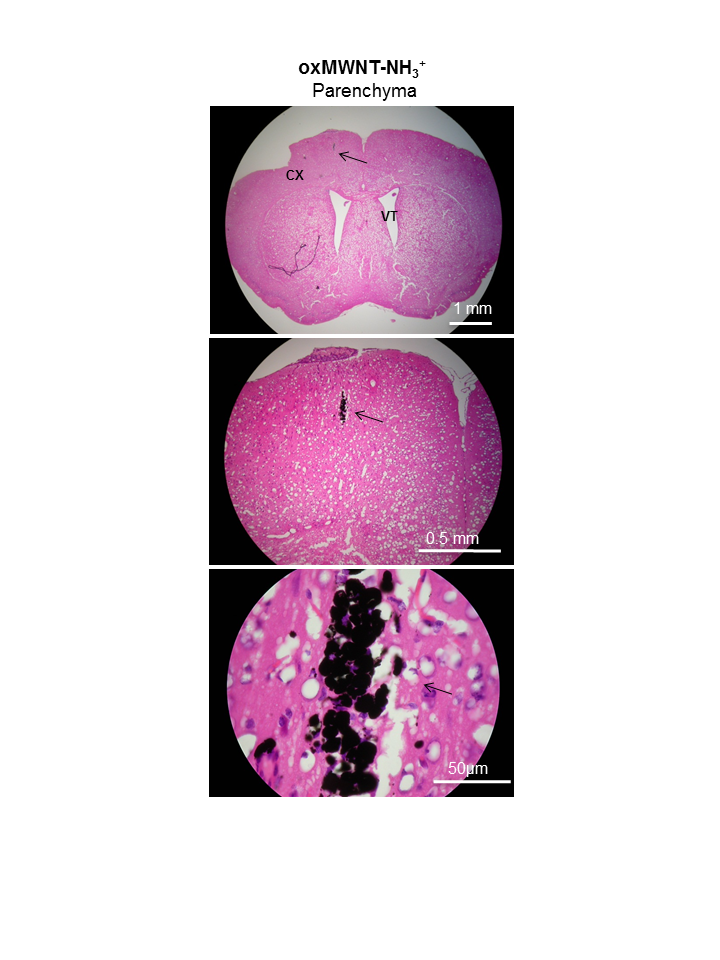

Supplement: Figure S1 — Brain distribution of oxMWNT−NH3+ after stereotactic administration in the motor cortex of C57BL/6 mice by light microscopy of H&E stained coronal sections, two weeks after injection with nanotubes. Whole coronal section of the brain (top) shows oxMWNT−NH3+ in the brain parenchyma. Middle and bottom panels show high magnification images of the oxMWNT−NH3+ detected in brain parenchyma. Cortex (CX), and ventricles (VT) are noted, respectively. Black arrows indicate the presence of f-MWNTs in the sections. (TIF) [file pone.0080964.s001.tif]

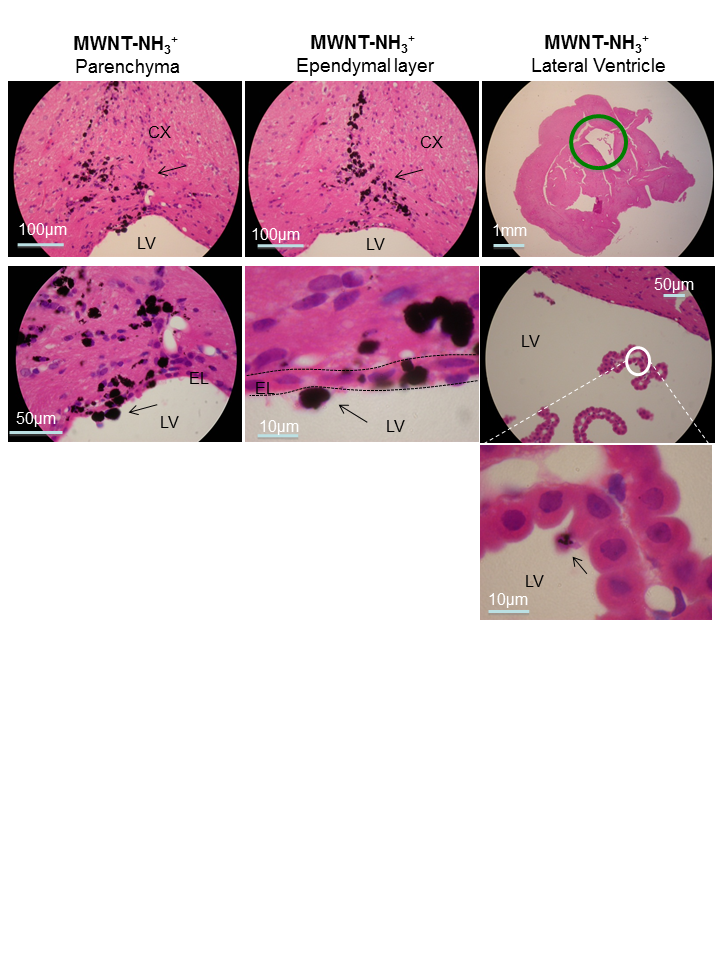

Supplement: Figure S2 — Brain distribution of MWNT−NH3+ after stereotactic administration in the motor cortex of C57BL/6 mice by light microscopy of H&E stained coronal sections, two weeks after injection with nanotubes. Parenchymal (left), Ependymal Layer (middle) and Lateral Ventricle (right) regions are shown, depicting the presence of MWNT−NH3+ throughout these regions. Bottom panels show high magnification images of the MWNT−NH3+ injected tissue. Cortex (CX), ependymal layer (EL), and ventricles (VT) are noted, respectively. Black arrows indicate the presence of f-MWNTs in the sections. (TIF) [file pone.0080964.s002.tif]

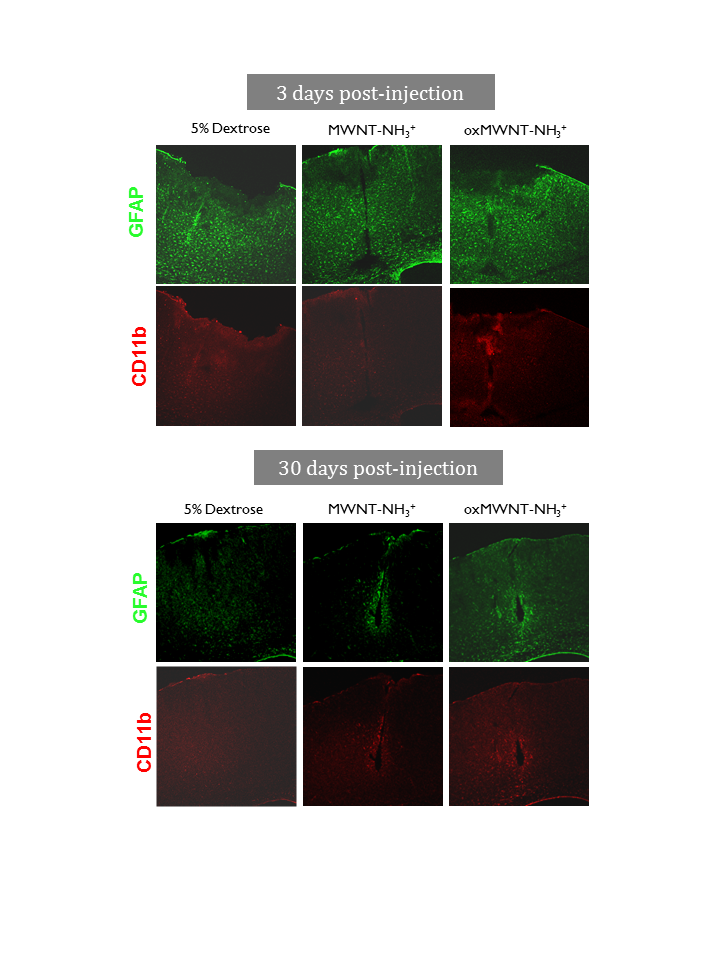

Supplement: Figure S4 — Glia activation studies after stereotactic injection. A) & B) Show immunohistochemical staining of brain sections after injection with 5% dextrose solution, MWNT−NH3+ and oxMWNT−NH3+, 3 and 30 days post-injection respectively. Green channel represents glial fibrillary acidic protein (GFAP) positive cells (astrocyte marker) and the red channel represents CD11b positive cells (microglia marker). (TIF) [file pone.0080964.s004.tif]

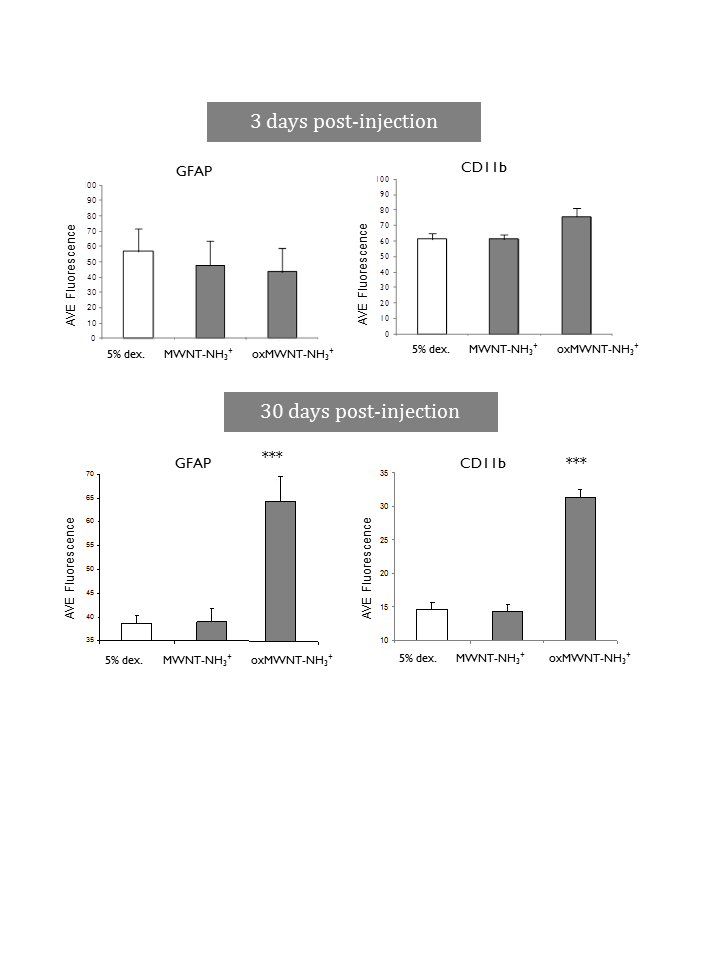

Supplement: Figure S5 — Glia activation studies after stereotactic injection with 5% dextrose saline solution, MWNT−NH3+ and oxMWNT−NH3+ (n = 3 for each group). Average fluorescence intensity corresponding to total glia (GFAP) and microglia (CD11b) activation after 3 and 30 days. The intensity was measured and quantified in an area 1×0.5 mm surrounding the injection site. (TIF) [file pone.0080964.s005.tif]

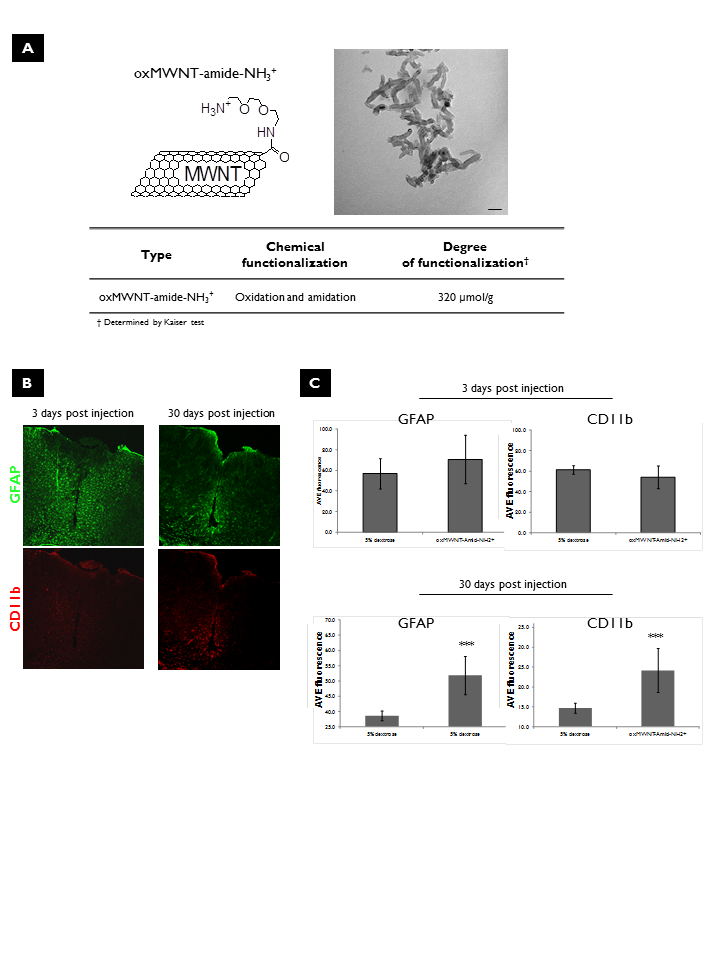

Supplement: Figure S6 — Effects from cortical injection with oxMWNT−amide−NH3+. A) Chemical structure and TEM images of oxMWNT−amide−NH3+ dispersed in 5% dextrose at 250µg/ml final concentration (scale bar 100 nm). C) & D) Glia activation studies after stereotactic injection with oxMWNT−amid−NH3+. C) Immunohistochemistry of brain sections at 3 and 30 days post-administration. Green channel represents GFAP-positive cells (total glia), red channel represents CD11b-positive cells (microglia). D) Average fluorescence intensity induced by astrocyte activation (GFAP) and by microglia activation (CD11b) after 3 and 30 days, respectively. Intensity was measured and quantified in an area of 1×0.5 mm surrounding the injection sites. (TIF) [file pone.0080964.s006.tif]

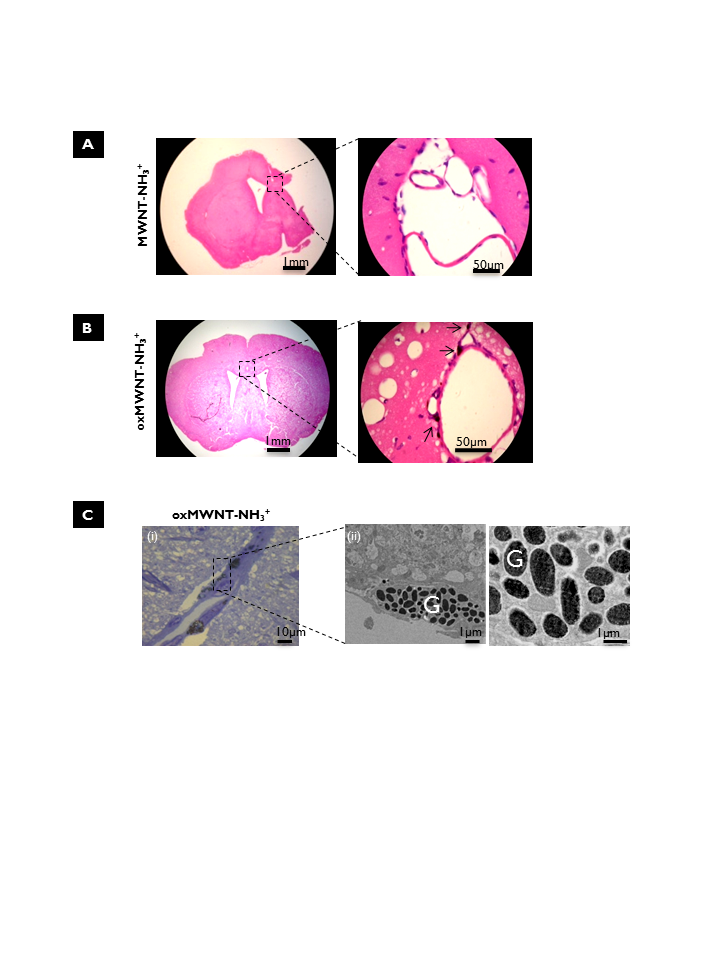

Supplement: Figure S7 — H&E stained light microscopy images of brain sections 14 days post-injections with: A) MWNT−NH3+ and B) oxMWNT−NH3+. H&E staining shows no evidence of the presence of mast cells after stereotactic injection of MWNT−NH3+. Mast cells (arrows) could be visualized after injection of oxMWNT−NH3+ (B and C); C) shows semi-thin section of brain parenchyma 14 days post –injection with oxMWNT−NH3+ stained with toluene blue. Ci) & Cii) represent TEM of mast cells with their typical granules (G) present in the cytoplasm. (TIF) [file pone.0080964.s007.tif]
